# Broad autism phenotype and parental personality in parents of preschoolers with autism: associations with social and behavioral profile

**DOI:** 10.3389/frcha.2026.1842230

**Published:** 2026-07-06

**Authors:** Francesco Craig, Filippo Muratori, Raffaella Devescovi, Giovanna Tritto, Giuseppina Palermo, Marco Turi

**Affiliations:** 1 Department of Cultures, Education and Society, Calabria University, Rende, Italy; 2 Department of Developmental Neuroscience, IRCCS Fondazione Stella Maris, Pisa, Italy; 3 Stella Maris Mediterraneo Foundation, Chiaromonte, Italy; 4 Department of Human and Social Sciences, Salento University, Lecce, Italy

**Keywords:** adaptive functioning, autism spectrum disorder, broader autism phenotype, parental personality, preschool children

## Abstract

**Introduction:**

Biological parents of children with Autism Spectrum Disorder (ASD) frequently exhibit features of the broader autism phenotype (BAP), encompassing subclinical social, cognitive, and personality traits related to autism. Elevated BAP rates among first-degree relatives support the strong heritability of ASD. This study investigated BAP traits and maladaptive personality domains in biological mothers and fathers of preschool-aged children at first ASD diagnosis and examined associations between parental BAP and children's autism features.

**Methods:**

Eighty-nine children with ASD (2–5 years) and their parents were assessed. Autism-related traits were measured using the Social Responsiveness Scale–Second Edition (SRS-2) and degree of autism features with the Autism Diagnostic Observation Schedule–Second Edition (ADOS-2). Parental personality functioning was evaluated with the Personality Inventory for DSM-5 (PID-5). Children's behavioral functioning and adaptive skills were assessed using the Child Behavior Checklist (CBCL 1.5–5) and Vineland Adaptive Behavior Scales–Second Edition (VABS-2). BAP was defined as an SRS-2T-score ≥60. Families were classified as BAP+ if at least one parent met this threshold and BAP− otherwise.

**Results:**

Twenty-five percent of parents met criteria for BAP (26% of fathers; 24% of mothers). Parental BAP was associated with higher maladaptive personality traits (all PID-5 domains). Children in BAP+ families showed higher SRS-2 total and subdomain scores and higher ADOS-2 CSS (*p* < .05), indicating greater degree of autism features. No group differences emerged in behavioral problems or adaptive functioning. Hierarchical regression analyses revealed that fathers' SRS-2 scores significantly predicted children's ADOS-2 domain scores after controlling for demographic and cognitive variables, whereas mothers' scores did not.

**Conclusions:**

Parental BAP traits, particularly in fathers, are associated with greater autism features in young children, underscoring the importance of intergenerational trait profiles for early assessment and family-based intervention planning.

## Introduction

Autism Spectrum Disorder (ASD) is a neurodevelopmental condition characterized by difficulties in social communication and interaction across various situations, along with the presence of restricted and repetitive behaviors, interests, or activities (1). The exact cause of ASD remains unclear. Evidence indicates that both genetic and environmental factors contribute to its development (2). Genetic mutations and syndromes explain about 10%–20% of cases (3), and heritability estimates range from 64% to 91% (4, 5), underscoring strong gene–environment interactions (6–9).

Furthermore, genetic, neurofunctional, and clinical evidence indicates that traits resembling, but less severe than, those of ASD are also heritable (10, 11). These subclinical features, often observed in first- and second-degree relatives of individuals with ASD, are described as the broad autism phenotype (12, 13). The BAP (Broader Autism Phenotype) reflects the etiological complexity of ASD and offers insight into the inheritance of autism-related traits (14). It encompasses subtle characteristics such as social communication and interaction differences, pragmatic language difficulties (15), cognitive rigidity (16), and repetitive behaviors (17).

Research consistently shows that the BAP is significantly more prevalent among parents of children with autism compared to the general population (18, 19). However, prevalence estimates vary widely, ranging from 2.6% to 80% in fathers, 3% to 52% in mothers, and 5.3% to 56% in both parents (16, 17, 20), reflecting substantial variability in the expression of subclinical traits within this phenotype. A higher presence of BAP has been observed in males (21–23), consistent with the broader male predominance seen in autism diagnoses (24). Children of parents exhibiting BAP are more likely to present repetitive behaviors as well as mild language and motor delays accompanied by emotional dysregulation (20), suggesting a potential link between parental BAP and both the inheritance and manifestation of autism-related traits in offspring (25). Importantly, several studies have documented familiarity and intergenerational transmission of these traits, suggesting that BAP characteristics may be heritable and contribute to the transmission of autism-related features across generations (26–30).

Early studies on BAP primarily relied on qualitative family assessments but gradually evolved to include structured psychometric tools. Initially, BAP traits were categorized dichotomously; however, since the 2000s, the construct has been increasingly viewed as a dimensional and continuous phenomenon rather than a binary trait (12). This dimensional perspective aligns with the concept of the autism spectrum, framing BAP as a continuum of autistic-like characteristics found both in the general population and among biological relatives of autistic individuals. Consequently, standardized psychometric scales have been developed to quantify these traits more accurately. Recently, Narzisi and colleagues (31) reported that 29% of parents of autistic children met criteria for BAP, with fathers showing higher Autism Quotient (AQ) scores than mothers. Paternal AQ scores were positively correlated with those of their children, emphasizing the potential role of paternal traits in shaping the expression of autism-related behaviors in offspring. However, the authors themselves acknowledge that the AQ may not fully capture the complexity of BAP traits and they recommend incorporating multi-informant approaches and complementary instruments, such as the Social Responsiveness Scale (SRS). Uljarević and colleagues (32) analyzed over 2.700 family triads using only the social motivation subscale of the SRS-2 and found that lower parental social motivation was independently and cumulatively associated with reduced social motivation in children with ASD.

Together, these findings highlight that BAP traits, particularly in social communication and motivation domains, represent a heritable and dimensional continuum rather than a categorical condition. The present study extends the previous research by applying the SRS-2 to explore the expression of BAP traits in parents of children with varying degrees of autism features. Unlike categorical diagnostic instruments, the SRS-2 provides a quantitative assessment of multiple dimensions, including social awareness, social cognition, social communication, social motivation, and restricted or repetitive behaviors. This multidimensional approach allows for the identification of specific socio-communicative profiles that may contribute to the intergenerational transmission and variability of autism-related features. Understanding how parental BAP influences developmental outcomes offers valuable insight into autism's variability and inheritance patterns and supports the development of parent-centered and early-intervention strategies tailored to the specific socio-cognitive profiles of both parents and children.

Another relevant aspect identified in the literature is the relationship between BAP traits and personality domains. Autistic-like traits and BAP traits should not be interpreted as manifestations of personality disorders; rather, they represent autism-related characteristics that may partially overlap with personality domains and, in some individuals, may co-occur with specific personality features or related psychopathology (33, 34). Research indicates that individuals with ASD show a distinct personality profile characterized by low Extraversion, Agreeableness, Conscientiousness, and Openness, and high Neuroticism (34, 35). Neuroticism was the strongest predictor distinguishing ASD from typically developing individuals. Similar patterns have been observed in non-clinical populations, where higher autistic traits correlate with greater Neuroticism and lower Extraversion, alongside poorer social and communication skills and stronger attention to detail (36, 37). A study by Li and colleagues (38) found that specific parental personality traits—such as rigidity, low openness, and high aggressiveness—were associated with greater social, behavioral, and adaptive difficulties in children with ASD, suggesting that parental personality profiles, possibly linked to the broader autism phenotype, may influence symptom expression in offspring.

In the present study, BAP traits were conceptualized as subclinical autism-related traits rather than as general personality traits. Previous research has shown associations between autism-related traits and personality domains, while also suggesting that these traits represent a distinct dimension (37). Moreover, although both BAP and personality traits show relative temporal stability, stability alone does not imply conceptual equivalence (39).

Although previous research has documented the presence and heritability of BAP traits in parents of autistic children, important gaps remain. First, less is known about how specific parental BAP dimensions relate to children's social-communicative, adaptive, and behavioral profiles. Second, the extent to which parental BAP traits are associated with personality domains in relatives of autistic individuals remains insufficiently understood. Addressing these gaps may help clarify how subclinical autism-related traits are expressed within families and how they relate to both child developmental outcomes and parental psychological profiles. The present study addresses these issues by using a multidimensional assessment of parental BAP traits and examining their associations with child outcomes and parental personality domains.

The primary objective of this study was to examine BAP traits in parents of preschool-aged children with an ASD diagnosis, using the SRS-2 as a measure of subclinical autism-related traits in the parents themselves. Based on this framework, we expected parental BAP traits to be most strongly associated with child characteristics that map onto the core autism phenotype, specifically the Social Affect and, to a lesser extent, the Restricted and Repetitive Behaviour domains as measured by the ADOS-2. This expectation is consistent with the conceptualization of the BAP as a subclinical expression of autism-related traits in first-degree relatives, involving social-communication differences, pragmatic language difficulties, rigidity, and aloofness (17, 40). Therefore, we hypothesized that parental BAP status would be primarily related to children's autism-specific socio-communicative features, rather than to broader indices of functioning such as adaptive behavior or emotional–behavioral problems. Furthermore, we expected fathers to exhibit higher levels of BAP traits than mothers.

Since few studies have analyzed the relationship between BAP and personality domains, this study also sought to investigate which specific personality facets are linked to the presence of BAP in parents. We hypothesized that parents exhibiting higher levels of BAP would demonstrate more pronounced deviations in personality trait profiles. Studying this association is important because it may clarify how subclinical autistic traits interact with personality dimensions, offering insight into the psychological and genetic mechanisms underlying the BAP. Understanding these relationships can help identify familial patterns of vulnerability or resilience, refine phenotypic characterization in autism research, and targeted support for families showing BAP-related personality profiles.

## Materials and methods

### Participants

The study involved a sample of 89 preschoolers with their first clinical diagnosis of ASD and their parents (Table 1). Children aged between 24 and 60 months (with a mean age of 42.30 ± 8.24 months), with non-verbal cognitive functioning (NVIQ) scores ranging from 20 to 120 (mean NVIQ 45.32 ± 25.54). Of these, 25 were female (28%), with the remainder being male (*n* = 64, 72%). Participants were recruited from a public secondary referral center for ASD in Southern Italy, specifically from Child and Adolescent Neuropsychiatry Unit, Ospedale Madonna delle Grazie and Fondazione Stella Maris Mediterraneo, Matera. This service functions as a second-level diagnostic unit receiving referrals from primary care pediatricians, community neuropsychiatric services, and rehabilitation centers for children requiring comprehensive neurodevelopmental assessment. Consequently, the population evaluated at this center tends to include a relatively high proportion of preschoolers with significant developmental delays, language impairments, and complex clinical presentations. All children had a primary diagnosis of ASD, with no other known medical or psychiatric comorbidities, except for speech disorders, regulation disorders, and anxiety traits. Diagnoses were established based on DSM-5 criteria (1). A multidisciplinary team, consisting of a senior child psychiatrist and an experienced research child psychologist, was responsible for performing the diagnosis. To confirm the clinical diagnosis, we used the Autism Diagnostic Observation Schedule, Second Edition (ADOS-2) (41), which is considered the gold-standard semi-structured instrument for observing and assessing communication abilities, social interaction, play quality, and imagination in children. Considering parents, the inclusion criteria were having a child with ASD aged between 2 and 6 years and being biological parents. To ensure consistency and completeness in the assessment of parental variables, only families in which both biological parents participated and completed all study measures were included in the present analyses. A total of 178 biological parents were included: 89 fathers (mean age 37.1 ± 6.47 years; age range 20 to 52 years) and 89 mothers (mean age 33.5 ± 5.64 years; range age 19 to 48 years). All participants were resident in Italy. The study adhered to ethical principles and the Declaration of Helsinki guidelines, with written informed consent obtained from parents or guardians prior to completing the questionnaire.

**Table 1:** Demographic and clinical characteristics of children with ASD and parental age in the total sample.

Demographic and clinical variables	Children (*n* = 89)
Gender	25 F: 64 M
Age (months)	
M (SD)	42.30 (8.24)
Range	24–60
NVIQ	
M (SD)	45.32 (25.54)
Range	20–120
CSS-Social Affect ADOS	
M (SD)	7.31 (1.91)
Range	3–10
CSS-RRB ADOS	
M (SD)	6.51 (2.01)
Range	1–10
CSS- ADOS Total Score	
M (SD)	7.09 (2.00)
Range	3–10
SRS-2 Total Score	
M (SD)	78.36 (12.56)
Range	45–90
Maternal Age (years)	
M (SD)	33.5 (5.57)
Range	19–48
Paternal Age (years)	
M (SD)	37.1 (6.47)
Range	20–52

NVIQ, non-verbal cognitive functioning; CSS, Calibrated Severity Score from the Autism Diagnostic Observation Schedule–Second Edition; SRS-2, Social Responsiveness Scale–Second Edition.

### Procedures and measures

The clinical assessment included a battery of standardized instruments administered to children and parents to evaluate multiple developmental and clinical domains. Children were assessed for non-verbal cognitive functioning using a measure selected according to each child's developmental and language profile (see Non-verbal Cognitive Assessment section), the ADOS-2, the Child Behavior Checklist (CBCL 1½–5), the Vineland Adaptive Behavior Scales–Second Edition (Vineland-2), and the Social Responsiveness Scale–Second Edition (SRS-2; preschool version). Adults were evaluated using the SRS-2 (adult self-report version) and the Personality Inventory for DSM-5–Brief Form (PID-5-BF).

### Non-verbal cognitive assessment

Due to heterogeneity in developmental and language profiles, a clinically derived estimate of non-verbal cognitive functioning (NVIQ) was obtained using different standardized instruments depending on each child's developmental and language profile. Specifically, the Performance IQ from the Wechsler Preschool and Primary Scale of Intelligence (WPPSI-III) (42), the Nonverbal IQ composite score from the Leiter International Performance Scale–Third Edition (Leiter-3) (43), and Developmental Quotient derived from the “Foundations of Learning” subscale of the Griffiths Mental Development Scales–Third Edition (GMDS-3) (44) were used. Scores obtained from these instruments were used as alternative indicators of non-verbal cognitive functioning and entered into a single study variable, with each child contributing one score derived from the instrument considered most appropriate for their developmental and language profile. This approach is consistent with precedents in the literature on preschool ASD samples (45). Approximately 42 children (47% of the sample) were assessed with the GMDS-III, typically those with more severe developmental delays for whom standard IQ measures were not applicable. As a consequence, the reported mean NVIQ should be interpreted as an estimate of non-verbal cognitive functioning and the lower average likely reflects both the severity profile of this subsample and differences in the nature of the cognitive indices provided by the instruments.

### Autism diagnostic observation scale-2

The ADOS-2 is a standardized, semi-structured observational instrument extensively used in the clinical evaluation of ASD. It provides a systematic framework for assessing core autism-related behaviors, including social communication, reciprocal interaction, and play, and is therefore considered a reference measure in both clinical and research contexts. The ADOS-2 includes multiple modules specifically designed to accommodate individuals of different ages and expressive language levels, enabling its application across a wide developmental range, from early childhood through adulthood. The selection of the appropriate ADOS-2 module is determined by the individual's chronological age and language abilities. Calibrated Severity Scores (CSS) were derived for each participant based on ADOS total scores and domain scores for Social Affect (SA) and Restricted and Repetitive Behaviors (RRB), following established algorithms (46–48). CSS range from 1 to 10 and allow comparison across different ADOS modules and versions. Moreover, CSS provide an index of degree of autism features that is relatively independent of age and language level, making them more suitable than raw ADOS scores for assessing symptom expression (49). For participants assessed with the ADOS-2 Toddler Module, total and domain CSS were calculated according to the procedures described by Esler et al. (50), allowing direct comparability with calibrated scores derived from other ADOS-2 modules.

### CBCL 1.5-5

The CBCL 1½–5 (51), which is widely used to evaluate children's behavior, was used in this study. The questionnaire involves parents rating a child's behaviors using a three-point scale, with 0 meaning “not true”, 1 meaning “somewhat or sometimes true”, and 2 meaning “very true or often true”. The CBCL generates scores for seven syndrome scales, three summary scales, and five DSM-Oriented scales (DOS). Clinical significance is defined as a T-score of 64 or higher for summary scales and a T-score of 70 or higher for syndrome and DOS. A borderline clinical range is indicated by values between 60 and 63 for summary scales or between 65 and 69 for syndrome and DOS. For the CBCL 1½–5, T-scores ≥64 on the summary scales are considered clinically significant, whereas scores between 60 and 63 fall within the borderline range. For syndrome and DSM-oriented scales, T-scores ≥70 are considered clinically significant, whereas scores between 65 and 69 indicate the borderline range. The questionnaire has been widely used for screening (52–54) and to investigate psychiatric comorbidity and emotion regulation (55–57) in preschoolers with ASD.

### Vineland adaptive behavior scales

Adaptive functioning was assessed using the Vineland-2 (58), a standardized instrument administered by a trained examiner through a semi-structured interview with parents or primary caregivers. The Vineland-II provides a comprehensive evaluation of everyday adaptive skills across four domains: Communication, encompassing receptive, expressive, and written abilities; Daily Living Skills, including personal, domestic, and community functioning; Socialization, which covers interpersonal relationships, play and leisure activities, and coping skills; and Motor Skills, assessing both gross and fine motor abilities. For each domain, as well as for the Adaptive Behavior Composite (ABC), standard scores are derived with a normative mean of 100 and a standard deviation of 15. The Vineland-2 has demonstrated strong psychometric properties, including good reliability and validity (58). In the present study, all adaptive functioning measures were derived from the Italian adaptation of the Vineland-2 (59), which has been previously employed in research involving preschool children with ASD (60). Scores from all Vineland-2 domains were included in the analyses.

### Social responsive scale-2 (adult and preschool version)

The SRS-2 (61) is a 65-item questionnaire designed to assess autism-related social impairments dimensionally. Items are rated on a 4-point Likert scale ranging from not true to almost always true. The SRS-2 yields five domain scores—Social Awareness, Social Cognition, Social Communication, Social Motivation, and Restricted Interests and Repetitive Behaviors (RRB)—as well as composite scores for Social Communication and Interaction (SCI) and RRB, and a Total score reflecting overall autism-related symptomatology. In the present study, parental BAP traits were assessed using the adult self-report version of the SRS-2, whereas children's autism-related social difficulties were evaluated using the preschool version, completed by the mother. Higher scores indicate greater degree of social impairment and autism-related traits. Raw scores were converted to standardized T-scores based on normative data (*M* = 50, SD = 10), allowing comparison across individuals and domains. SRS-2 scores are categorized as *T* = 59 or lower indicating typical functioning, *T* = 60–65 representing mild impairment, *T* = 66–75 indicating moderate impairment, and *T* = 76 or higher indicating severe impairment. The SRS-2 has demonstrated excellent psychometric properties across age groups and informants. Previous studies have reported high internal consistency for the adult version in neurotypical sample, with Cronbach's alpha values ranging from.94 to.96 for the Total score (62), as well as strong internal consistency for the preschool version (63). These findings support the reliability of the SRS-2 as a measure of both subclinical autism-related traits in adults and higher degree of autism features in young children.

### Personality inventory for DSM-5-brief form

The PID-5-BF (64) is a 25-item self-report screening instrument designed to assess maladaptive personality traits as conceptualized within the Alternative Model for Personality Disorders of the DSM-5 (1). The PID-5-BF measures five broad maladaptive trait domains: Negative Affectivity (e.g., emotional lability, anxiousness, separation insecurity, depressivity), Detachment (e.g., withdrawal, intimacy avoidance, anhedonia), Antagonism (e.g., manipulativeness, deceitfulness, grandiosity, callousness), Disinhibition (e.g., impulsivity, irresponsibility, distractibility, risk taking), and Psychoticism (e.g., unusual beliefs and experiences, eccentricity, cognitive and perceptual dysregulation). Each domain is assessed by five items rated on a 4-point Likert-type scale ranging from very false or often false to very true or often true. Higher mean scores indicate greater levels of maladaptive personality functioning within each trait domain. The PID-5-BF has demonstrated good psychometric properties in both adult and adolescent samples, supporting its use as a brief dimensional screening measure of maladaptive personality traits (65–68). The Italian version of the PID-5-BF has shown adequate internal consistency, with Cronbach's alpha coefficients ranging from approximately.70 to.86 across domains, as well as good construct validity (67).

### Data analysis

All statistical analyses were conducted using standard parametric procedures. Descriptive statistics were first computed for all study variables. Differences between mothers and fathers in age and autism-related traits, as measured by the SRS-2, were examined using independent-samples t-tests, with effect sizes estimated using Cohen's d. Parental BAP status was defined using the SRS-2 Total T-score, applying the standard “mild range” cutoff (*T* ≥ 60) to classify parents as BAP-positive (BAP+) or BAP-negative (BAP−). At the family level, families were classified as BAP+ when at least one biological parent met the BAP threshold, and as BAP− when neither parent reached the cutoff. To further characterize the broader autism phenotype in parents, we examined the association between BAP status and personality traits. A multivariate analysis of variance (MANOVA) was conducted on the five PID-5 personality domains, with BAP status (BAP+ vs BAP−) and gender (mothers vs. fathers) entered as between-subjects factors. Significant multivariate effects were followed by univariate analyses of variance, and effect sizes were reported using eta squared (*η*^2^).

To examine the impact of parental BAP status on children's clinical profiles, group differences between BAP+ and BAP− children were assessed on demographic variables (age, NVIQ, and gender distribution) using one-way analyses of variance (ANOVA) for continuous variables and chi-square tests for categorical variables. Subsequent one-way ANOVAs were conducted to compare groups on autism-related traits (SRS-2), clinician-rated degree of autism features (ADOS-2 CSS), emotional–behavioral functioning (CBCL 1.5–5), and adaptive functioning (VABS-2). Effect sizes were reported using eta squared (*η*^2^).

To further investigate the unique and incremental contribution of parental BAP traits to children's degree of autism features, hierarchical multiple regression analyses were performed. Separate regression models were estimated for each ADOS-2 outcome variable (Total CSS, Social Affect CSS, and RRB CSS). In all models, children's age and non-verbal IQ were entered as control variables in the first step. Fathers' SRS-2 Total T-scores were entered in the second step to assess the incremental contribution of paternal BAP traits. Mothers' SRS-2 Total T-scores were entered in the third step to evaluate whether maternal BAP traits explained additional variance beyond child-related factors and paternal BAP traits. Changes in explained variance (*Δ*R^2^) were examined at each step to assess the relative contribution of predictors. Statistical significance was set at *p* < 0.05 (two-tailed) for all analyses.

## Results

### Comparison of maternal and paternal characteristics (broader autism phenotype)

Before examining associations between parental BAP traits and children's clinical profiles, we first compared maternal and paternal characteristics. Mothers and fathers did not differ in their levels of autism-related traits as measured by the SRS-2 as shown by Table 2. Mean SRS Total scores were highly comparable between mothers and fathers, and no significant differences emerged across any of the SRS-2 domain T-scores (all *p* > 0.10), except for a small difference in the Social Motivation domain (*p* = 0.036). These results indicate that both parents exhibited similar levels of subclinical autism-related traits.

**Table 2:** Comparison of maternal and paternal autism-related traits (SRS-2) and age.

Parental variables	Fathers (n 89) M ± SD	Mothers (n 89) M ± SD	Groups comparison	*p*	Effect Size
Age (years)	37.1 ± 6.47	33.5 ± 5.64	t_(176)_ = −4.00	0.001	*d* = 0.6
SRS-2 Social Awareness	56.0 ± 8.56	55.2 ± 7.85	t_(176)_ = −0.621	0.535	*d* = −0.09
SRS-2 Social Cognition	54.6 ± 8.93	56.7 ± 9.55	t_(176)_ = 1.564	0.120	*d* = 0.23
SRS-2 Social Communication	57.2 ± 9.78	55.8 ± 8.97	t_(176)_ = −1.030	0.304	*d* = 0.15
SRS-2 Social Motivation	52.4 ± 8.45	55.2 ± 9.16	t_(176)_ = 2.118	**0**.**036**	*d* = 0.31
SRS-2 RRB	51.8 ± 7.37	53.6 ± 8.43	t_(176)_ = 1.467	0.144	*d* = 0.21
SRS-2 SCI Composite	56.3 ± 8.91	57.0 ± 9.07	t_(176)_ = 0.567	0.571	*d* = 0.08
SRS-2 Total Score	55.3 ± 8.67	56.3 ± 8.95	t_(176)_ = 0.791	0.430	*d* = 0.11

SRS-2, Social Responsive Scale-Second edition. Significant comparisons are highlighted in bold (*p* < 0.05).

Following the standard “mild range” threshold recommended by Constantino (61) and applied in Rubenstein et al. (20), a T-score ≥ 60 was used to identify parents exhibiting broader autism phenotype characteristics (BAP+). Based on this criterion, families were divided into two groups: a BAP+ group, which included all families in which at least one biological parent met the BAP threshold, and a BAP− group, comprising families in which neither parent reached the cutoff. This classification allowed us to test whether the presence of BAP traits in parents was associated with differences in their personality traits. After classification 23 (26%) fathers and 22 (24%) mothers were included in BAP+ groups. A multivariate analysis of variance was conducted on the five PID-5 domains (Negative Affectivity, Detachment, Antagonism, Disinhibition, and Psychoticism), with BAP status (BAP+ vs. BAP−) and gender (mothers vs. fathers) as between-subjects factors. As shown in Table 3 the multivariate analysis revealed a significant main effect of BAP status across all multivariate test statistics (all *p* < 0.001), indicating that parents classified as BAP+ differed from BAP− parents in their overall personality profile. A significant multivariate effect of gender was also observed (*p* = 0.002), whereas the BAP × gender interaction was not significant at the multivariate level (*p* = 0.319). Follow-up univariate analyses showed that BAP+ parents scored higher than BAP− parents across all PID-5 domains, including Negative Affectivity (*p* = 0.002), Detachment (*p* < 0.001), Antagonism (*p* < 0.001), Disinhibition (*p* < 0.001), and Psychoticism (*p* < 0.001). With respect to gender differences, mothers showed higher levels of Negative Affectivity than fathers, whereas fathers exhibited higher levels of Antagonism across both BAP groups (*p* = 0.007 and *p* = 0.025, respectively). No significant gender differences were observed in the other domains. A significant interaction between BAP and gender emerged for the Detachment domain (*p* = 0.025). Inspection of group means indicated that this interaction was driven by higher Detachment scores in fathers relative to mothers within the BAP+ group, whereas minimal gender differences were observed in the BAP− group.

**Table 3:** Descriptive statistics and univariate effects for PID-5 personality domains as a function of parental BAP status and gender.

PID-5 Domain	BAP− Mothers Mean (SD)	BAP− Fathers Mean (SD)	BAP+ Mothers Mean (SD)	BAP+ Fathers Mean (SD)	BAP Effect (1,174)	Gender Effect (1,174)	BAP × Gender (1,174)
Negative Affectivity	1.03 (0.69)	0.75 (0.63)	1.27 (0.72)	1.15 (0.70)	*F* = 9.91, *p* = .002, *η*^2^ = .054	*F* = 7.45, *p* = .007, *η*^2^ = .041	*F* = 0.18, *p* = .673, *η*^2^ = .001
Detachment	0.52 (0.49)	0.41 (0.44)	0.80 (0.57)	1.03 (0.62)	*F* = 19.83, *p* < .001, η^2^ = .101	*F* = 2.31, *p* = .130, η^2^ = .013	*F* = 5.12, *p* = .025, η^2^ = .029
Antagonism	0.16 (0.28)	0.27 (0.34)	0.38 (0.41)	0.49 (0.46)	*F* = 18.46, *p* < .001, η^2^ = .095	*F* = 5.13, *p* = .025, η^2^ = .029	*F* = 0.44, *p* = .507, η^2^ = .003
Disinhibition	0.45 (0.46)	0.45 (0.43)	0.72 (0.55)	0.86 (0.60)	*F* = 21.07, *p* < .001, η^2^ = .106	*F* = 1.88, *p* = .172, η^2^ = .011	*F* = 0.92, *p* = .339, η^2^ = .005
Psychoticism	0.28 (0.38)	0.21 (0.34)	0.57 (0.52)	0.58 (0.54)	*F* = 20.41, *p* < .001, η^2^ = .103	*F* = 0.64, *p* = .425, η^2^ = .004	*F* = 0.02, *p* = .887, η^2^ < .001

PID-5 = Personality Inventory for DSM-5. BAP− = neither parent met the SRS-2 BAP threshold; BAP+ = at least one parent met the threshold. η^2^ = eta squared (proportion of total variance explained by each effect). Univariate effects follow significant multivariate MANOVA results. *p* < .05, *p* < .01, *p* < .00.

### Child autism features as a function of parental BAP status

This parental BAP status classification allowed us to test whether the presence of BAP traits in the parental dyad—irrespective of whether exhibited by the mother, the father, or both—was associated with differences in children's degree of autism features, behavioral functioning, and adaptive skills. After classification 37 children (41% of the sample) comprised BAP+ group and 52 children (59% of the sample) were in BAP− group. As shown in Table 4, children in the BAP+ group consistently exhibited higher degree of autism features across both parent-report and clinician-rated measures. First, the two groups of children did not differ with respect to age (*p* = 0.17), NVIQ (*p* = 0.78), or gender distribution (*p* = 0.50), indicating that they were comparable on key demographic and cognitive variables. On the SRS-2, significant group differences emerged in multiple domains. Children with BAP+ parents showed higher scores in Social Communication (*p* = 0.010), Social Motivation (*p* = 0.003), and Restricted and Repetitive Behaviors (*p* = 0.048). The Social Communication and Interaction (SCI) composite was also significantly elevated in the BAP+ group (*p* = 0.013). Although Social Awareness and Social Cognition, showed only a trend (respectively *p* = 0.05 and *p* = 0.055), the overall pattern indicated more pronounced socio-communicative difficulties in children of BAP+ parents. Consistently, the SRS-2 Total score was significantly higher among BAP+ children compared to BAP− children (*p* = 0.009). These findings were corroborated by the ADOS-2. Children in the BAP+ group showed significantly higher clinician-rated degree of autism features on both the Total CSS (*p* = 0.002) and the Social Affect CSS (*p* = 0.002). The RRB CSS showed a non-significant trend in the same direction (*p* = 0.126). Together, these results indicate that parental BAP+ status is associated with a more pronounced autism phenotype in children, particularly in the social communication and social affective domains.

**Table 4:** Child autism features, cognitive functioning, and demographic characteristics as a function of parental BAP status.

Demographic and Clinical Variables	BAP+ (n 37) M ± SD	BAP− (n 52) M ± SD	Groups comparison	*p*	Effect Size
Gender	M: 28 F: 9	M: 36 F: 16	X_(1)_ = 0.44	0.505	phi = 0.07
NVIQ	42.57 ± 22.38	45.42 ± 22.56	F_(1,87)_ = 0.08	1.877	*η*^2^ = 0.001
Age (months)	40.89 ± 7.98	43.31 ± 8.35	F_(1,87)_ = 1.88	0.173	η^2^ = 0.02
SRS-2 Social Awareness	71.49 ± 11.35	66.46 ± 12.45	F_(1,87)_ = 3.79	0.055	η^2^ = 0.04
SRS-2 Social Cognition	78.76 ± 12.28	72.87 ± 14.71	F_(1,87)_ = 3.96	0.050	η^2^ = 0.04
SRS-2 Social Communication	81.14 ± 8.79	74.37 ± 13.86	F_(1,87)_ = 6.85	**0**.**010**	η^2^ = 0.07
SRS-2 Social Motivation	75.11 ± 11.14	66.62 ± 14.07	F_(1,87)_ = 9.31	**0**.**003**	η^2^ = 0.09
SRS-2 RRB	80.19 ± 13.20	74.35 ± 13.77	F_(1,87)_ = 4.03	**0**.**048**	η^2^ = 0.04
SRS-2 SCI Composite	81.03 ± 9.95	74.23 ± 13.95	F_(1,87)_ = 6.44	**0**.**013**	η^2^ = 0.07
SRS-2 Total Score	82.43 ± 9.25	75.46 ± 13.83	F_(1,87)_ = 7.12	**0**.**009**	η^2^ = 0.07
ADOS-2 CSS Total	7.84 ± 1.76	6.56 ± 2.01	F_(1,87)_ = 9.70	**0**.**002**	η^2^ = 0.10
ADOS-2 CSS Social Affect	8.05 ± 1.87	6.79 ± 1.77	F_(1,87)_ = 10.51	**0**.**002**	η^2^ = 0.11
ADOS-2 CSS RRB	6.89 ± 1.94	6.23 ± 2.03	F_(1,87)_ = 2.38	0.126	η^2^ = 0.02

NVIQ, non-verbal cognitive functioning; ADOS-2, Autism Diagnostic Observation Scale-2; SRS-2, Social Responsive Scale-Second edition. Significant comparisons are highlighted in bold (*p* < 0.05).

### Adaptive functioning and behavioural problems as a function of parental BAP status

To examine whether parental BAP status was associated with children's adaptive skills and emotional–behavioral functioning, we compared VABS-2 and CBCL 1.5–5 scores between the BAP+ and BAP− groups (Supplementary Table S1). ANOVA revealed no significant group differences on any CBCL scale, including internalizing and externalizing problems and DOS scores. Similarly, no differences were observed between groups across VABS-2 domains—Communication, Daily Living Skills, Socialization, and Motor Skills—or on the Adaptive Behavior Composite (all *p* > .05). These results indicate that, despite the presence of significant group differences in degree of autism features, children of BAP+ and BAP− parents showed comparable levels of adaptive functioning and emotional–behavioral problems.

### Parental BAP traits as predictors of degree of autism features

To examine the relative and incremental contribution of parental BAP traits to children's degree of autism features, three hierarchical multiple regression analyses were conducted with ADOS-2 Total CSS, Social Affect CSS, and RRB CSS as dependent variables. In all models, children's age and NVIQ were entered in the first step, followed by fathers' SRS-2 Total scores in the second step and mothers' SRS-2 Total scores in the third step. As shown in Table 5, across models, NVIQ emerged as a significant negative predictor of both ADOS-2 Total CSS (*β* = −0.018, *p* = 0.028) and Social Affect CSS (*β* = −0.023, *p* = 0.002), indicating higher clinician-rated degree of autism features in children with lower non-verbal cognitive functioning, whereas age did not significantly contribute to any outcome. Importantly, the inclusion of fathers' SRS-2 Total scores in the second step resulted in a significant increase in explained variance for all three ADOS-2 outcomes, with paternal BAP traits consistently predicting higher clinician-rated autism features across Total (*β* = 0.071, *p* = 0.004), Social Affect (*β* = 0.045, *p* = 0.049), and RRB (*β* = 0.074, *p* = 0.003) domains. In contrast, the addition of mothers' SRS-2 Total scores in the final step did not lead to a further increase in explained variance in any model, and maternal BAP traits did not emerge as significant predictors (all *p* > 0.05). To verify that combining scores from different cognitive instruments did not bias the regression findings, we additionally reran all three hierarchical models separately for the two cognitive-assessment subgroups to examine whether combining scores from different cognitive instruments influenced the regression findings. The paternal effect showed no divergent or opposing pattern across subgroups, although its statistical significance varied by ADOS-2 domain; maternal SRS-2 scores were consistently non-significant across all subgroup models (see Supplementary Tables S2, S3).

**Table 5:** Hierarchical multiple regressions model across ados-2 domains.

Model	Predictor	R^2^	R^2 change^	F^change^	df	*p*
Hierarchical multiple regressions – Dependent variable: **ADOS-2 CSS Total**						
Step 1	Age & NVIQ	0.053	–	2.366	86	0.100
Step 2	**Father SRS-2 Total Score**	**0**.**148**	**0**.**095**	**9**.**408**	**85**	**0**.**003**
Step 3	Mother SRS-2 Total Score	0.148	0.012	0	84	0.914
Hierarchical multiple regressions – Dependent variable: **ADOS-2 CSS Social Affect**						
Step 1	**Age & NVIQ**	**0**.**128**	**–**	**6**.**258**	**86**	**0**.**003**
Step 2	**Father SRS-2 Total Score**	**0**.**173**	**0**.**04**	**4**.**490**	**85**	**0**.**037**
Step 3	Mother SRS-2 Total Score	0.174	0.001	0.133	84	0.716
Hierarchical multiple regressions – Dependent variable: **ADOS-2 CSS RRB**						
Step 1	Age & NVIQ	0.012	–	0.517	86	0.598
Step 2	**Father SRS-2 Total Score**	**0**.**107**	**0**.**095**	**8**.**911**	**85**	**0**.**004**
Step 3	Mother SRS-2 Total Score	0.112	0.006	0.524	84	0.471

Predictors were tested in separate regressions models, controlling variables were entered as a block in the first step. NVIQ, non-verbal cognitive functioning; ADOS-2, Autism Diagnostic Observation Scale-2; SRS-2, Social Responsive Scale-Second edition. Statistically significant results are highlighted in bold (*p* < 0.05).

## Discussion

The present study investigated the expression of the BAP in parents of preschool-aged children with autism and examined its associations with maladaptive personality facets in parents and their children's clinical profiles at the time of first diagnosis. By adopting a dimensional approach and combining parent-report and clinician-rated measures, this study contributes to a more nuanced understanding of intergenerational patterns underlying autism-related traits.

Consistent with previous literature, a substantial proportion of parents met criteria for the BAP when applying the SRS-2 mild-range cutoff, supporting the notion that subclinical autistic traits are relatively common among first-degree relatives of autistic individuals (12, 17, 31). The prevalence of BAP observed in the present sample is broadly comparable to rates reported in other countries. For instance, Bora and colleagues (69) reported BAP traits in 25.2% of fathers and 17.8% of mothers of autistic children, while Wheelwright and colleagues (70) found similar proportions in a UK sample (33% of fathers and 23% of mothers). In a US study, Maxwell and colleagues (71) reported lower prevalence rates, particularly among mothers (21% of fathers and 10% of mothers), suggesting some cross-sample variability in the expression of parental BAP traits. Taken together, these findings indicate that while the prevalence of parental BAP is consistently elevated across studies, its exact distribution across mothers and fathers may vary depending on sample characteristics, assessment instruments, and cultural or methodological factors.

A systematic review of prevalence estimates has shown that the BAP is generally more prevalent in fathers than in mothers of children with autism, supporting the notion of a sex-related gradient in the expression of subclinical autistic traits (17). However, contrary to our initial hypothesis, mothers and fathers in the present study did not differ substantially in their overall levels of autism-related traits, as measured by the SRS-2, with the exception of a small but statistically significant difference in the Social Motivation domain. This pattern is consistent with previous studies that directly assessed BAP traits in both parents and reported largely comparable levels between mothers and fathers across most domains (72–76). Together, these findings suggest that gender differences in the BAP may be subtle and domain-specific rather than global, particularly when autism-related traits are assessed using dimensional, quantitative instruments such as the SRS-2 (32, 61). Further research using other measures of BAP with a sample of parents of children with autism is needed to confirm this finding, given the conflicting evidence coming from literature. These results underscore the potential value of systematically assessing parental BAP traits during first diagnostic evaluations, particularly in fathers. Parental autistic traits may provide useful contextual information during screening and family-informed assessment, particularly in identifying families in which autism-related traits may be more broadly expressed. Incorporating brief, standardized measures of parental BAP, such as the SRS-2, may therefore complement routine assessments without substantially increasing assessment burden.

In a neurodiversity-informed framework, the presence of BAP traits in parents can be understood not only as an index of familial liability, but also as an expression of neurocognitive variation that is continuously distributed in the general population. This interpretation aligns with the dimensional approach adopted in the present study and underscores the importance of distinguishing between individual differences in social-communicative style and clinically significant impairment, which may emerge when such traits interact with environmental demands and available supports. From this perspective, identifying BAP traits may help characterize family profiles and developmental contexts without automatically construing these characteristics as inherently maladaptive.

A novel contribution of the present study concerns the relationship between BAP status and maladaptive personality domains. Parents classified as having a BAP exhibited a broad pattern of elevated maladaptive personality dimensions across all PID-5 domains, including Negative Affectivity, Detachment, Antagonism, Disinhibition, and Psychoticism. These results extend previous evidence linking autistic traits to specific personality profiles in both clinical and non-clinical populations (34–37, 77), suggesting that the BAP may be embedded within a wider constellation of personality-related vulnerabilities. Importantly, while both BAP status and gender independently contributed to personality variation, interaction effects were limited, indicating that BAP-related personality differences are largely consistent across mothers and fathers, except for Detachment, which appeared particularly elevated in fathers within the BAP. The association between BAP and specific personality traits has important implications for parent-centered care. Elevated traits such as detachment, negative affectivity, and disinhibition may influence parents' emotional availability, stress regulation, and engagement with clinical services. In families in which one or both parents exhibit BAP-related personality vulnerabilities, clinicians may encounter challenges related to communication, alliance-building, or treatment adherence. Recognizing these trait profiles may support more tailored psychoeducational approaches, improve therapeutic engagement, and reduce the risk of misunderstanding or attrition during early intervention.

At the child level, parental BAP status was associated with higher degree of autism features, particularly in socio-communicative domains. Children of BAP+ parents showed higher autism-related features across both parent-reported and clinician-rated measures, especially in the Social Affect domain. These findings are consistent with prior evidence indicating familial aggregation and intergenerational transmission of autism-related traits (20, 30, 32) and support the hypothesis that parental BAP traits may contribute to the phenotypic variability observed in children with autism. Notably, group differences were specific to core autism features and were not accompanied by differences in adaptive functioning or emotional–behavioral problems, as measured by the VABS-2 and CBCL. This pattern suggests a selective association between parental BAP traits and autism-specific features, rather than a generalized increase in developmental, adaptive, or behavioral impairment. Clinically, this distinction is relevant because it indicates that, even when familial autism-related traits are present, children's adaptive functioning and emotional–behavioral adjustment may not be necessarily constrained by parental BAP status. These domains may therefore remain important targets for individualized intervention and environmental support, with the potential to modify developmental trajectories in children with ASD. It is important to acknowledge that these findings were observed in a sample characterized by very low to severely low non-verbal cognitive functioning, which may have influenced both the expression of autism features and their associations with parental BAP traits.

Hierarchical regression analyses further clarified the relative contribution of maternal and paternal BAP traits. After controlling for child age and non-verbal cognitive functioning, paternal, but not maternal, SRS-2 scores consistently predicted clinician-rated degree of autism features across ADOS-2 Total, Social Affect, and RRB domains. It is important to note that this is a differential predictive finding rather than a difference in trait levels. Mothers and fathers did not differ significantly in their overall BAP trait scores, and the paternal effect reflects a stronger predictive role in regression models rather than higher trait expression. This finding is particularly relevant because ADOS-2 scores were derived from direct clinician observation rather than from parental report, reducing the likelihood that the association merely reflects shared-informant bias.

The result is consistent with prior evidence suggesting that BAP traits may be more detectable or more strongly expressed in fathers of autistic children, and that paternal autistic traits may be associated with variation in offspring autism-related phenotypes (17, 31, 71, 75, 78).

Several, non-mutually exclusive mechanisms may account for this association involving paternal BAP traits. From a genetic perspective, paternal BAP traits may represent a marker of inherited liability for autism-related traits. Quantitative autistic traits show familial aggregation and continuity across generations, supporting the view that subclinical traits in parents may index genetic susceptibility contributing to variability in children's autism phenotypes (27, 30, 32, 73). In this sense, paternal BAP traits may not act as causal environmental factors *per se*, but rather as observable indicators of a familial liability that is expressed in both parent and child. A second possible explanation concerns sex-related and paternal biological mechanisms. Several studies have reported that BAP traits may be more prevalent, more strongly expressed, or more readily detectable in fathers than in mothers of autistic children, although findings remain somewhat heterogeneous across assessment methods and samples (17, 26, 75, 78). These patterns may reflect sex-related differences in the expression of autistic traits, threshold effects, or differences in measurement sensitivity. In addition, paternal contributions to neurodevelopmental outcomes may involve mechanisms beyond inherited common genetic variation, including *de novo* mutations, paternal age-related genetic risk, and epigenetic processes affecting the male germline (79–81). Finally, interactional pathways should also be considered. The BAP has been conceptualized as involving subclinical social-communicative differences, pragmatic language difficulties, rigidity, and aloofness or reduced social reciprocity, all of which may shape interpersonal functioning in family contexts (17). In line with this view, previous work has suggested that parental BAP traits may influence how parents engage with their children during social-communication interventions and parent–child exchanges (82). Moreover, evidence from family interaction studies indicates that BAP traits, particularly paternal BAP, may be associated with differences in observed relational quality, cooperation, and affective engagement within family interactions (83). Therefore, fathers with elevated BAP traits may show distinctive social-communicative styles, including reduced reciprocity, pragmatic difficulties, rigidity, or higher detachment, which could influence the quality and frequency of reciprocal social exchanges and children's exposure to complex social cues. This interpretation is also consistent with the present finding that BAP+ fathers showed particularly elevated Detachment scores, suggesting that paternal social-interactional style may be relevant for understanding child socio-communicative outcomes. More broadly, systematic reviews of father–child interactions in preschool children with ASD highlight that fathers remain underrepresented in autism research, despite evidence that father–child interactional patterns may contribute to children's social-communicative development and may represent an important target for individualized parent-mediated interventions (84).

However, given the cross-sectional design of the present study, these hypotheses remain speculative. Future studies should combine longitudinal designs, genetically informed family models, and direct observational measures of father–child and mother–child interactions to disentangle inherited liability from epigenetic and environmental pathways.

While maternal traits undoubtedly play a central role in early development and caregiving, the present findings suggest that paternal autism-related traits, as measured in the present study, showed a stronger predictive role for children's degree of autism features, suggesting that paternal contributions may warrant specific attention in future research, though this finding should be interpreted cautiously given the absence of significant group-level differences in BAP trait levels between parents. These results suggest the importance of including paternal BAP traits in models of intergenerational variability, while acknowledging that the mechanisms underlying this differential predictive pattern remain to be clarified.

In conclusion, these results underscore the importance of conceptualizing the broader autism phenotype as a multidimensional construct encompassing both autism-related traits and personality characteristics, with meaningful implications for child outcomes. From a clinical perspective, timely identification of BAP traits in parents—especially fathers—may help clinicians better contextualize variability in children's clinical presentation at diagnosis and tailor parent-mediated interventions accordingly. Psychoeducational and early-intervention programs that consider parental trait profiles may enhance engagement, communication, and long-term outcomes for autistic children and their families.

### Limitations and future directions

Several limitations of the present study should be acknowledged. First, the assessment of parental broader autism phenotype relied exclusively on self-report measures (SRS-2 Adult), which may be subject to reporting biases related to insight, self-awareness, or social desirability. Previous work has emphasized the importance of multi-informant and multimethod approaches in the assessment of BAP traits, including partner-report, clinician-rated instruments, and observational measures, to more fully capture the complexity of subclinical autism-related traits in adults (17, 75). Moreover, future research should examine the relationship between parents' self-perception of their own autism-related traits and potential clinical diagnoses, as this may influence how parents perceive, interpret, and respond to their child's difficulties. This approach could help inform more targeted awareness, psychoeducation, and family-centered intervention strategies. An additional limitation is that parental mental health was not directly assessed. Parents of autistic children are known to show elevated levels of anxiety, depressive symptoms, and parenting stress (85, 86), which may partially overlap with or amplify BAP-related characteristics. Future research should explicitly examine parental mental health as a potential moderator or mediator of the association between parental BAP traits and child degree of autism features.

Third, the sample size was relatively small, including 89 families. Although comparable to other clinically based studies, this may have limited statistical power to detect smaller effects or more complex interaction patterns, such as differential contributions of maternal-only vs. paternal-only BAP profiles. The modest sample size may also limit the generalizability of the findings. Replication in larger, independent, and more diverse samples is therefore warranted to confirm the robustness and clinical relevance of the present results. Beyond methodological considerations, the characteristics of the study sample also impose constraints on the interpretation of the findings. Although participants exhibited substantial variability in non-verbal cognitive functioning, the overall level of cognitive functioning was notably low. As detailed in the methods, this pattern likely reflects both the characteristics of the recruiting center, a secondary-level referral service that primarily evaluates children with significant developmental delays and complex neurodevelopmental presentations and the use of different measures of non-verbal functioning, including both IQ-based and developmental quotient index. Cognitive functioning is known to influence multiple aspects of development, including social communication, adaptive behavior, symptom presentation, and parent-reported outcomes. Although NVIQ was included as a covariate in all regression models, both the low average level and the substantial heterogeneity of cognitive functioning may have contributed to variability in the observed associations between parental BAP traits and child clinical characteristics. Consequently, the present findings should be interpreted with caution and may not generalize to autistic preschoolers with average or near-average cognitive functioning. Future studies should replicate these analyses in larger and more representative samples encompassing a broader range of cognitive abilities. Reassuringly, stratified regression analyses conducted separately for participants assessed with IQ-based instruments vs. the GMDS-III yielded a broadly consistent pattern of paternal prediction in both subgroups (Supplementary Tables S2, S3), suggesting that the NVIQ variable did not meaningfully distort the key regression findings.

Fourth, the cross-sectional design precludes conclusions regarding causal mechanisms underlying the observed associations between parental BAP traits and child degree of autism features. Longitudinal studies are needed to disentangle genetic transmission effects from early interactional processes and to examine how parental traits may shape developmental trajectories over time. A particularly important methodological concern is the potential for shared-method variance arising from overlapping informants. Specifically, mothers completed both their own SRS-2 self-report and the child SRS-2 proxy-report, which may have artificially inflated associations between maternal autism-related traits and child SRS-2 outcomes due to shared rater effects. Importantly, however, the ADOS-2-based findings are less susceptible to this bias, as they derive from independent clinician observation. The fact that paternal SRS-2 scores predicted children's ADOS-2 outcomes further reduces the likelihood that the key regression findings are attributable to shared-informant bias. Nonetheless, future research incorporating multiple informants and observational measures at the child level would further enhance the interpretability of intergenerational associations.

## Data Availability

The raw data supporting the conclusions of this article will be made available by the authors, without undue reservation.
