# Supplementary material for: Broad autism phenotype and parental personality in parents of preschoolers with autism: associations with social and behavioral profile
Source: Front Child Adolesc Psychiatry. 2026 Jul 6;5:1842230. doi: 10.3389/frcha.2026.1842230 (PMC13381743; doi:10.3389/frcha.2026.1842230)
Supplement: Supplementary file 1 [file Table1.docx]

|  | **BAP+ (n = 37) M ± SD** | **BAP– (n = 52) M ± SD** | **Groups comparison** | ***p*** | **Effect Size** |
| --- | --- | --- | --- | --- | --- |
| **VABS-2 Communication** | 49.4 ± 11.37 | 51.0 ± 15.93 | F(1,87)=0.25 | 0.618 | η² = 0.003 |
| **VABS-2**  **Daily Living Skills** | 61.1 ± 11.56 | 60.7 ± 12.59 | F(1,87)=0.02 | 0.888 | η² = 0.000 |
| **VABS-2**  **Socialization** | 60.8 ± 10.23 | 61.5 ± 12.89 | F(1,87)=0.08 | 0.776 | η² = 0.001 |
| **VABS-2**  **Motor Skills** | 69.9 ± 16.08 | 69.3 ± 12.22 | F(1,87)=0.04 | 0.850 | η² = 0.000 |
| **VABS-2**  **Adaptive Behavior Composite** | 57.4 ± 16.97 | 58.3 ± 17.02 | F(1,87)=0.06 | 0.808 | η² = 0.001 |
| **CBCL**  **Internalizing Problems** | 61.1 ± 7.11 | 57.3 ± 11.06 | F(1,87)=3.21 | 0.077 | η² = 0.036 |
| **CBCL**  **Externalizing Problems** | 55.2 ± 8.04 | 53.8 ± 10.46 | F(1,87)=0.48 | 0.491 | η² = 0.005 |
| **CBCL**  **Total Problems** | 58.0 ± 9.63 | 54.2 ± 11.33 | F(1,87)=2.72 | 0.103 | η² = 0.030 |
| **CBCL**  **AFF** | 59.1 ± 6.91 | 56.9 ± 7.92 | F(1,87)=1.85 | 0.178 | η² = 0.021 |
| **CBCL**  **ANX** | 56.1 ± 6.95 | 54.8 ± 6.83 | F(1,87)=0.88 | 0.352 | η² = 0.010 |
| **CBCL**  **PDP** | 69.6 ± 7.63 | 67.6 ± 10.39 | F(1,87)=0.99 | 0.323 | η² = 0.011 |
| **CBCL**  **ADHD** | 59.7 ± 7.42 | 57.9 ± 7.14 | F(1,87)=1.32 | 0.254 | η² = 0.015 |
| **CBCL**  **OPP** | 53.9 ± 5.44 | 54.6 ± 6.36 | F(1,87)=0.29 | 0.594 | η² = 0.003 |

**Table S1. Adaptive functioning and emotional–behavioral problems as a function of parental BAP status.**

VABS-2 = Vineland Adaptive Behavior Scales–Second Edition; CBCL = Child Behavior Checklist 1.5–5; AFF = Affective Problems; ANX = Anxiety Problems; PDP = Pervasive Developmental Problems; ADHD = Attention-Deficit/Hyperactivity Problems; OPP = Oppositional Defiant Problems. Significant comparisons are highlighted in bold (p < .05).

**Table S2. Hierarchical multiple regressions model across ADOS-2 domains in the subgroup assessed with a developmental instrument (GMDS; n=42).**

| Model | Predictor | R^2^ | R^2 change^ | F ^change^ | df | *p* |
| --- | --- | --- | --- | --- | --- | --- |
| Hierarchical multiple regressions – Dependent variable: **ADOS-2 CSS Total** | | | | | | |
| Step 1 | Age & NVIQ | 0.031 | -- | 0.621 | 39 | 0.543 |
| Step 2 | **Father SRS-2 Total Score** | **0.128** | **0.097** | **4.242** | **38** | **0.046** |
| Step 3 | Mother SRS-2 Total Score | 0.129 | 0.001 | 0.046 | 37 | 0.831 |
| Hierarchical multiple regressions – Dependent variable: **ADOS-2 CSS Social Affect** | | | | | | |
| Step 1 | Age & NVIQ | 0.066 | -- | 1.389 | 39 | 0.261 |
| Step 2 | Father SRS-2 Total Score | 0.088 | 0.021 | 0.886 | 38 | 0.352 |
| Step 3 | Mother SRS-2 Total Score | 0.088 | 0 | 0.012 | 37 | 0.912 |
| Hierarchical multiple regressions – Dependent variable: **ADOS-2 CSS RRB** | | | | | | |
| Step 1 | Age & NVIQ | 0.013 | -- | 0.253 | 39 | 0.778 |
| Step 2 | **Father SRS-2 Total Score** | **0.163** | **0.150** | **6.805** | **38** | **0.013** |
| Step 3 | Mother SRS-2 Total Score | 0.174 | 0.011 | 0.494 | 37 | 0.487 |

Predictors were tested in separate regressions models, controlling variables were entered as a block in the first step. NVIQ= non-verbal cognitive functioning; ADOS-2: Autism Diagnostic Observation Scale-2; SRS-2 =Social Responsive Scale-Second edition. Statistically significant results are highlighted in bold (p < 0.05).

**Table S3. Hierarchical multiple regressions model across ADOS-2 domains in the subgroup assesed with IQ-based instruments (Leiter-3 or WPPSI-III; n=47).**

| Model | Predictor | R^2^ | R^2 change^ | F ^change^ | df | *p* |
| --- | --- | --- | --- | --- | --- | --- |
| Hierarchical multiple regressions – Dependent variable: **ADOS-2 CSS Total** | | | | | | |
| **Step 1** | **Age & NVIQ** | 0.054 | -- | 1.251 | 44 | 0.296 |
| Step 2 | Father SRS-2 Total Score | **0.136** | **0.082** | **4.087** | **43** | **0.049** |
| Step 3 | Mother SRS-2 Total Score | 0.141 | 0.005 | 0.257 | 42 | 0.615 |
| Hierarchical multiple regressions – Dependent variable: **ADOS-2 CSS Social Affect** | | | | | | |
| Step 1 | **Age & NVIQ** | **0.171** | **--** | **4.549** | **44** | **0.016** |
| Step 2 | **Father SRS-2 Total Score** | **0.248** | **0.076** | **4.366** | **43** | **0.043** |
| Step 3 | Mother SRS-2 Total Score | 0.258 | 0.010 | 0.568 | 42 | 0.455 |
| Hierarchical multiple regressions – Dependent variable: **ADOS-2 CSS RRB** | | | | | | |
| Step 1 | Age & NVIQ | 0.013 | -- | 0.298 | 44 | 0.744 |
| Step 2 | Father SRS-2 Total Score | 0.046 | 0.033 | 1.477 | 43 | 0.231 |
| Step 3 | Mother SRS-2 Total Score | 0.047 | 0.001 | 0.030 | 42 | 0.864 |

Predictors were tested in separate regressions models, controlling variables were entered as a block in the first step. NVIQ= non-verbal cognitive functioning; ADOS-2: Autism Diagnostic Observation Scale-2; SRS-2 =Social Responsive Scale-Second edition. Statistically significant results are highlighted in bold (p < 0.05).
